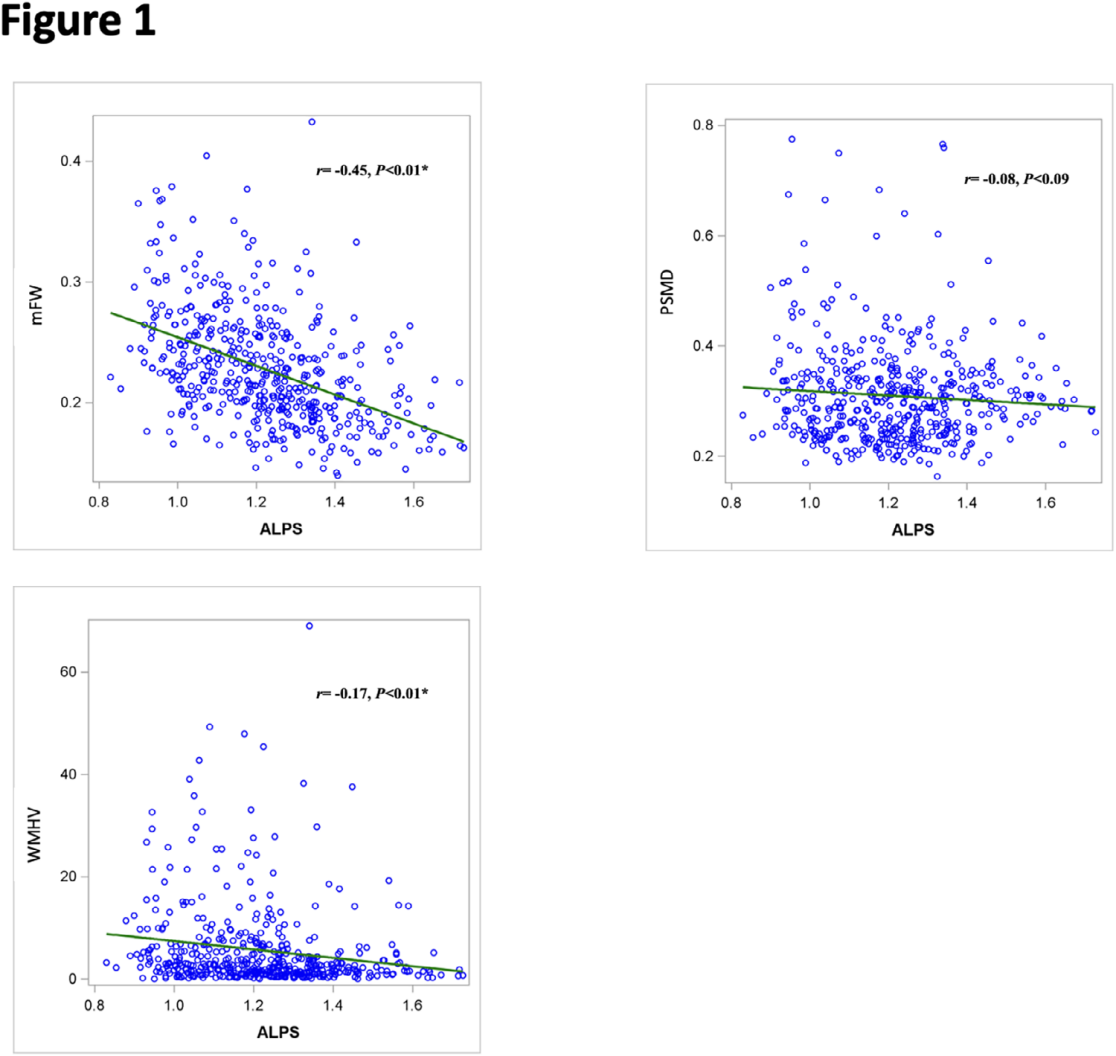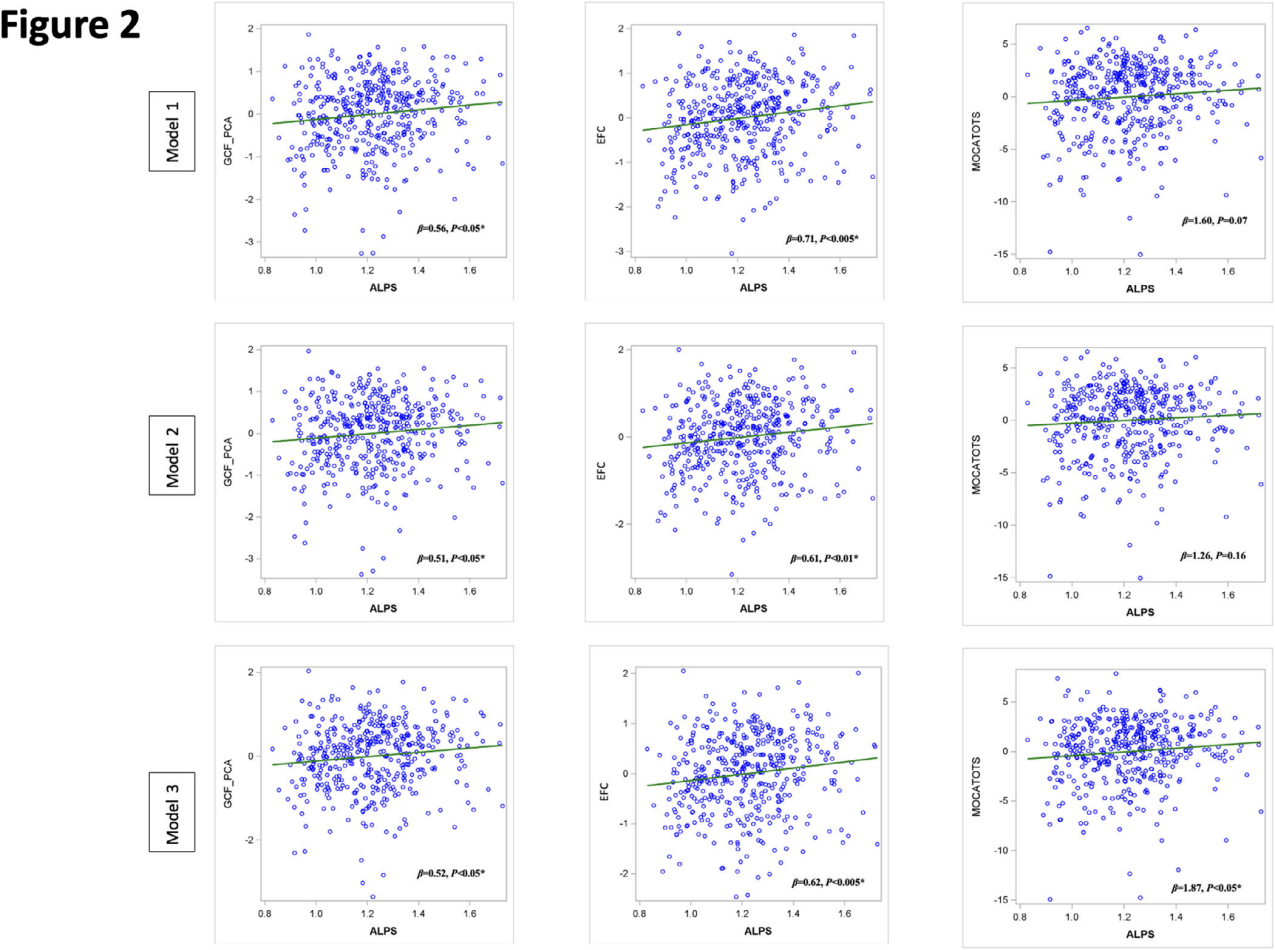# Validation of diffusivity along the perivascular space as a biomarker for vascular cognitive impairment and dementia

**DOI:** 10.1002/alz.084965

**Published:** 2025-01-09

**Authors:** Xiaodan Liu, Giuseppe Barisano, Pauline Maillard, Caprihan Arvind, Steven Cen, Xingfeng Shao, Kay Jann, John M Ringman, Hanzhang Lu, Konstantinos Arfanakis, Charles Decarli, Brian T Gold, Clandia L. Satizabal, Elyas Fadaee, Mohamad Habes, Lara Stables, Herpreet Singh, Andre J. van der Kouwe, Kristin Schwab, Karl G Helmer, Steven M. Greenberg, Danny JJ Wang, MarkVCID Consortium

**Affiliations:** ^1^ University of California, San Francisco, San Francisco, CA USA; ^2^ Laboratory of FMRI Technology (LOFT), Mark & Mary Stevens Neuroimaging and Informatics Institute, University of Southern California, Los Angeles, CA USA; ^3^ Zilkha Neurogenetic Institute, University of Southern California, Los Angeles, CA USA; ^4^ Stanford University, Stanford, CA USA; ^5^ Department of Neurology and Center for Neuroscience, University of California, Davis, CA USA; ^6^ The Mind Research Network, Albuquerque, NM USA; ^7^ Keck School of Medicine, University of Southern California, Los Angeles, CA USA; ^8^ University of Southern California, Los Angeles, CA USA; ^9^ Mark and Mary Stevens Neuroimaging and Informatics Institute, University of Southern California, Los Angeles, CA USA; ^10^ Memory and Aging Center, University of Southern California, Los Angeles, CA USA; ^11^ Johns Hopkins University School of Medicine, Baltimore, MD USA; ^12^ Illinois Institute of Technology, Chicago, IL USA; ^13^ Rush University Medical Center, Chicago, IL USA; ^14^ University of California Davis, Davis, CA USA; ^15^ University of Kentucky, Lexington, KY USA; ^16^ Population Health Sciences and Glenn Biggs Institute for Neurodegenerative Diseases, University of Texas Health Science Center at San Antonio, San Antonio, TX USA; ^17^ Neuroimage Analytics Laboratory and Biggs Institute Neuroimaging Core, Glenn Biggs Institute for Neurodegenerative Diseases, University of Texas Health Science Center at San Antonio, San Antonio, TX USA; ^18^ Neuroimage Analytics Laboratory and Glenn Biggs Institute Neuroimaging Core, Glenn Biggs Institute for Neurodegenerative Diseases, University of Texas Health San Antonio, San Antonio, TX USA; ^19^ Memory and Aging Center, UCSF Weill Institute for Neurosciences, University of California, San Francisco, San Francisco, CA USA; ^20^ Massachusetts General Hospital, Boston, MA USA; ^21^ Athinoula A. Martinos Center for Biomedical Imaging, Massachusetts General Hospital, Charlestown, MA USA; ^22^ Massachusetts Institute of Technology, Cambridge, MA USA; ^23^ Harvard Medical School, Boston, MA USA; ^24^ Athinoula A. Martinos Center for Biomedical Imaging, Massachusetts General Hospital, Harvard Medical School, Charlestown, MA USA

## Abstract

**Background:**

To validate the index of diffusivity along the perivascular space (ALPS index) as a biomarker for vascular cognitive impairment and dementia (VCID).

**Method:**

The participants and MRI data used in this study were acquired as part of the MarkVCID consortium, which consisted of seven sites. A total of 578 participants (72.5±7.2 years old, 232 Male) who received baseline and follow‐up cognitive evaluations (Montreal Cognitive Assessment (MoCA), Principal Component Analysis derived General Cognitive Function (GCF_PCA), and composite score of Executive Function (EFC)) and MRI examinations were included in this study. The diffusion tensor imaging (DTI) data were processed by using an in‐house automatic processing pipeline with FMRIB Software Library 6.0.6. The mean free water (mFW) and peak width of skeletonized mean diffusivity (PSMD) were computed in the white matter (WM). The ALPS index was defined as the average of bilateral ALPS indices which were calculated by the ratio of mean of x‐axis diffusivity in the projection fibers (Dxxproj) and x‐axis diffusivity in the association fibers (Dxxassoc) to the mean of y‐axis diffusivity in the projection fibers (Dyyproj) and z‐axis diffusivity in the association fibers (Dzzassoc). The WM hyperintensity volumes (WMHV) were calculated on FLAIR images and normalized by intracranial volume (ICV). Univariate correlation (Pearson or Spearman) was used to examine the associations between imaging markers (ALPS index, mFW, PSMD, and WMHV). Linear regression models were used to evaluate the associations of baseline ALPS index with baseline and longitudinal changes of cognitive outcomes, regressing out three types of covariates: 1) age, sex, and education, 2) added vascular risk factors (VRFs), including diabetes, hypertension and smoking, 3) further added mFW, PSMD and WMHV. SAS 9.4 software was used for all statistical analyses, and P<0.05 was regarded as statistical significance.

**Result:**

The baseline ALPS index was significantly correlated with existing biomarkers of cerebral small vessel disease (cSVD)‐related VCID, i.e., mFW and WMHV (P<0.01) (Figure 1), and baseline cognitive performances, i.e., MoCA total score, GCF_PCA score, and EFC score (P<0.05) after adjusting for the demographics, VRFs, and existing biomarkers (Figure 2).

**Conclusion:**

the ALPS index is an independent contributor to the cognitive decline in cSVD.